# Is the One-Compartment Model with First Order Absorption a Useful Approximation?

**DOI:** 10.1007/s11095-023-03582-1

**Published:** 2023-08-18

**Authors:** Michael Weiss

**Affiliations:** Department of Pharmacology, Martin Luther University Halle-Wittenberg, Halle, Germany

**Keywords:** bateman function, inverse gaussian function, model misspecification, population pharmacokinetics

## Abstract

**Purpose:**

The one-compartment model with first order absorption (ka1C) has been extensively used to fit oral data. But when the disposition parameters of the drug are not available, the bias in the parameter estimates remains unclear. In this paper, the effect of potential misspecification of the area under the curve (*AUC*) and the mean absorption time (*MAT*) was evaluated for three relatively slowly absorbed drugs/formulations.

**Methods:**

Assuming a three-compartment disposition model with an input (absorption) rate described as a sum of two inverse Gaussian functions (2IG3C) as the true model, the deviations of *AUC* and *MAT* estimated with simpler models were analyzed. Simpler models, as the ka1C model (Bateman function), the one-compartment model with IG input function (IG1C) and the gamma density function were fitted to the oral data alone, and compared to the fits obtained with the 2IG3C model which also uses the 3C disposition parameters of the drug. Data from pharmacokinetic studies of trospium, propiverine and ketamine in healthy volunteers were analyzed using a population approach.

**Results:**

The Bateman function (ka1C) allowed a robust estimation of the population mean *AUC*, but the individual estimates were highly biased. It failed in evaluating *MAT*. The simple alternative models did not improve the situation.

**Conclusions:**

The Bateman function appears to be useful for estimating the population mean value of *AUC* after oral administration. The results reemphasize the fact that insight into the absorption process can be only gained when also intravenous reference data are available.

**Supplementary Information:**

The online version contains supplementary material available at 10.1007/s11095-023-03582-1.

## Introduction

Just 100 years ago Murray Lyon published a paper entitled “The absorption of adrenalin” [1] where he derived the biexponential function for the first time in pharmacology. Dost was probably not aware of this work when he applied this model (later known as Bateman function) to oral absorption [2]. Now it is the most popular model in pharmacokinetics and has been used in more than 3000 population pharmacokinetic studies. The reason lies in the apparent simplicity of this model. It implies, however, two unrealistic assumptions:1) that the maximum of the absorption rate is reached immediately and 2) that the drug distributes instantaneously throughout the body (one - compartment model). Furthermore, it was argued that this would lead to unphysiologically long absorption times [3]. Nevertheless it may be a useful approximation for fitting oral concentration-time data when no information on distribution kinetics is available (i.e., no iv reference data). But according to the motto “Seek simplicity and distrust it.” [4], one should be aware of the approximations made and the potential effect of model misspecification.

Thus, the purpose of this communication is to examine the questions 1) of whether useful information on the absorption process can be extracted solely from data after oral (po) administration and 2) whether the Bateman function can serve as an empirical model to fit oral data. To this end, results obtained by using the Bateman function (monoexponential absorption rate) are compared with those obtained when fitting po data to a more complex model based on a flexible time course of absorption rate together with iv disposition parameters. Since this model not only provided an optimal fit (in contrast to the Bateman function) but also uses realistic assumptions, the parameter estimates were taken as the “true” parameter values. In the same way, we analyzed the performance of improvements of the one-compartment model, replacing the first order input function (absorption rate constant *k*_*a*_) by a flexible input function, i.e. a time- dependent fractional absorption rate *k*_*a*_(*t*) [5, 6]. Finally, the usefulness of the empirical alternative model, the unimodal gamma function [7] was investigated.

The model independent parameters area under the curve (*AUC*) and mean absorption time (*MAT*) resulting from the different alternative approaches were compared with the “true” values showing the differences in both population means and individual subject parameters. One specific question was whether reasonable estimates of *MAT* can be obtained from oral data alone. Note that we use the term *MAT* originally proposed by Cutler [8] as a measure of rate of bioavailability; it denotes the average time it takes for molecules to enter the systemic circulation following oral administration.

The study is based on bioavailability data of three drugs that were previously analyzed by the absorption model mentioned above. Data from a trospium study [9] and those obtained for the lowest and highest doses in the studies of propiverine [10] and R-ketamine [11] were analyzed. Propiverine and ketamine were administered as extended release and trospium as an immediated release formulation. Propiverine, ketamine are rapidly absorbed while trospium is only slowly absorbed. The low bioavailability of ketamine is due to a substantial hepatic first-pass extraction.

## Methods

### Model Based on Oral and Intravenous Reference Data (True Model)

The data analysis has been described in detail in the original publications cited above. In short, the data obtained after iv injection were first fitted using a 3-compartment model; then, holding the six disposition parameters fixed, the parameters of the input function, i.e. the time course of the absorption rate (rate of drug input into the central compartment), *I*(*t*), were estimated by fitting the oral data. *I*(*t*) was described as a sum of two sum of inverse Gaussian (IG) functions (2IG3C model).

1 $$I(t)= DF\left({pf}_1(t)+\left(1-p\right){f}_1(t)\right)\kern0.5em 0<p<1$$

where *D* is dose, *F* is bioavailability, *f*_*i*_(*t*) denotes the IG function below and *p* is a nonnegative quantity that defines the relative contribution of each IG to the input function *I*(*t*).

2 $${f}_i(t)=\sqrt{\frac{M{T}_i}{2\pi \kern0.1em R{D}_i^2\kern0.20em {t}^3}}\exp \left[-\frac{{\left(t-M{T}_i\right)}^2}{2\kern0.1em R{D}_i^2\kern0.20em M{T}_it}\right],t>0$$

where *MT*_*i*_ and ${RD}_i^2$ are the scale and shape parameters, respectively, of the *i*th IG function. The mean absorption time is then given by

3 $$MAT= pM{T}_1+\left(1-p\right)M{T}_2$$

and $$AUC=FD/CL$$.

where *CL* denotes clearance.

In the literature, one can find many examples where this model was successfully applied [12–16]. Since *k*_*a*_ is not anymore a constant as for first-order absorption, but rather a function of time, we introduce the time-dependent absorption rate coefficient or fractional absorption rate *k*_*a*_(*t*) [5].

4 $$\frac{d{A}_{gi}}{dt}=-{k}_a(t){A}_{gi}(t)$$

where *A*_*gi*_ denotes the unabsorbed drug amount (note that *dA*_*gi*_/*dt* = *I*(*t*), Eq. 1).

### Models Based Solely on Oral Data

Assuming that no iv reference data are available, the following models were used to fit oral plasma concentration-time data to:

1. One-compartment model with first order absorption (ka1C)

The result is the Bateman function with absorption and elimination rate constants, *k*_*a*_ and *k*_*e*_,

5 $$C(t)=A\left({e}^{-{k}_et}-{e}^{-{k}_at}\right)$$

Where *MAT* = 1/*k*_*a*_ and

6 $$AUC=\frac{A\left({k}_a-{k}_e\right)}{k_a{k}_e}$$

2. One-compartment model with IG input function (IG1C)

Instead of assuming a monoexponential absorption rate, i.e., a constant fractional absorption rate *k*_*a*_, the time course of absorption rate is described by a single IG function.

$$I\left(t\right)=DFf\left(t\right)$$

with *f*(*t*) given by Eq. 2. Then *k*_*a*_ becomes a time-dependent function *k*_*a*_(*t*), and

$${\displaystyle \begin{array}{c} MAT= MT\\ {} AUC= FD/ CL\end{array}}$$*.*

3. Unimodal gamma curve [7]

6 $$\begin{array}{cc}C(t)={At}^{a-1}e^{-bt}&a>1\end{array}$$

7 $$AUC=\frac{A\Gamma (a)}{b^a}$$

where Γ is the gamma function. Note that here no estimate of *MAT* is available.

### Parameter Estimation

Parameter estimation was performed by population analysis (nonlinear mixed-effects modeling) using the ADAPT (Version 5) software [17]. ADAPT 5 provides estimates of the population mean and inter-subject variability as well as of the individual subject parameters (conditional means). We assumed log-normally distributed model parameters and that the measurement error has a standard deviation that is a linear function of the measured quantity. ‘Goodness of ft’ was assessed using the Akaike information criterion (AIC) and by plotting the predicted *versus* the measured responses. In all cases “rich” po data were fitted: Trospium 12 subjects with 24 measurements per subject, propiverine 10 subjects with 16 measurements per subject and ketamine 15 subjects with 14 measurements per subject. The mean plasma concentration time curves can be found in the Supplementary Material.

## Results

**Fig. 1:**
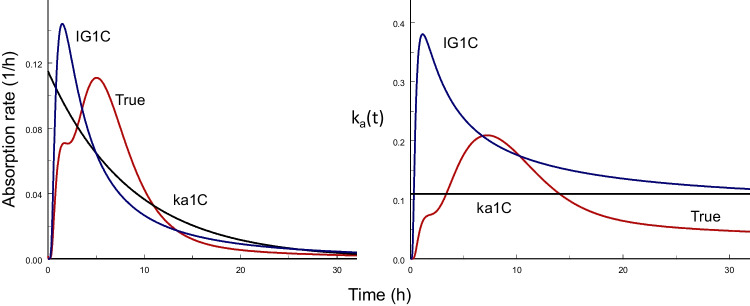
Dose normalized time profiles of absorption rates resulting from fitting po data to the Bateman function (ka1C), to the IG input and one-compartment disposition model (IG1C) (both without iv data), and to the 2IG3C model with iv data (true model) (True) (left), and the corresponding time-dependent absorption rate coefficients (right). The curves were simulated using the population mean parameter estimates of the input function.

That the essential difference between the approaches lays in the models of the absorption process is shown in Fig. 1, where the time courses of absorption rates and of time-dependent absorption rate coefficients, *k*_*a*_(*t*), are shown for trospium as an example. It is clear *a priori* that the absorption rate increases from 0 to a maximum, and cannot be maximal at time 0 as for the ka1C model. The IG1C model reaches its (higher) maximum too early. The corresponding fractional absorption rates vary with time, except for ka1C model where *k*_*a*_ = const. The *k*_*a*_(*t*)-function of the IG1C model reaches this value asymptotically.

**Fig. 2:**
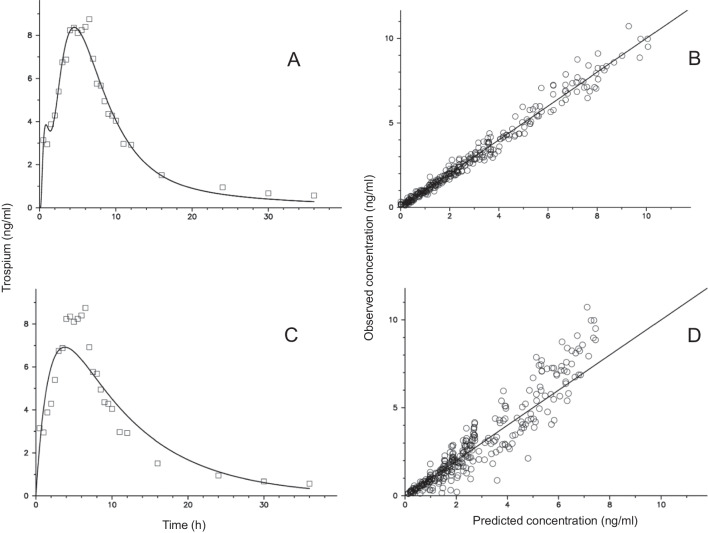
Examples of fits of trospium data for a subject to the 2IG3C model with iv data (true model) (**A**) and to the ka1C model (Bateman function) (**C**). The corresponding goodness-of-fit plots (observed concentration *versus* the individual model-predicted values for all 12 subjects) are shown on the right site (**B** and **D**).

In no case an individual *C*(*t*) curve could be adequately fitted to a Bateman function (ka1C). This is also due to the fact that no concentration-time curve was log-concave, i.e., the logarithm of *C(t*) was not a concave function of time [5, 15]. A comparison of the goodness of fit plots is shown for trospium in Fig. 2. The AIC values for the models are summarized in Table I. The fractional deviations of the population mean values of *AUC* and *MAT* from those of the reference model (true values) are depicted in Table II. For *AUC* the relative low biases of the population means are in contrast to the large biases in the individual estimates (Fig. 2). The deviations of the *MAT* mean values estimated with the ka1C and IG1C model are unacceptable high for all drugs (except for trospium) (Table II).

**Table I:** AIC Values for the 2IG Input Model with fixed Disposition Parameters (2IG3C, True Model), the one Compartment Model with First Order Input (ka1C), the one Compartment Model with IG Input (IG1C) and the Gamma Curve (Gamma)

Model	Trospium (n = 12)	Propiverine (n = 10)		Ketamine (n = 15)	
	30 mg^a^	10 mg^a^	45 mg^a^	10 mg^a^	80 mg^a^
2IG3C	274	−1222	−847	−287	396
ka1C	618	−1009	−701	−208	752
IG1C	458	−1114	945	−111	436
Gamma	882	733	1129	6	912

^a^Oral dose

**Table II:** Percent Deviation of the Parameters Estimated with the ka1C Model (Bateman Function), the IG1C Model and the Gamma Model from the True Values (2IG Input Model with fixed Disposition Parameters)

Model	Population	Trospium (n = 12)	Propiverine (n = 10)		Ketamine (n = 15)	
	mean	30 mg^a^	10 mg^a^	45 mg^a^	10 mg^a^	80 mg^a^
ka1C	AUC	2.8	−1.5	−0.8	11	−0.7
	MAT	−5.9	−50	−70	−56	−44
IG1C	AUC	1.9	−0.5	−0.4	−6.3	−2.3
	MAT	13	67	−69	−58	−17
Gamma	AUC	8.7	−7	−10	20	1.8

^a^Oral dose

## Discussion

**Fig. 3:**
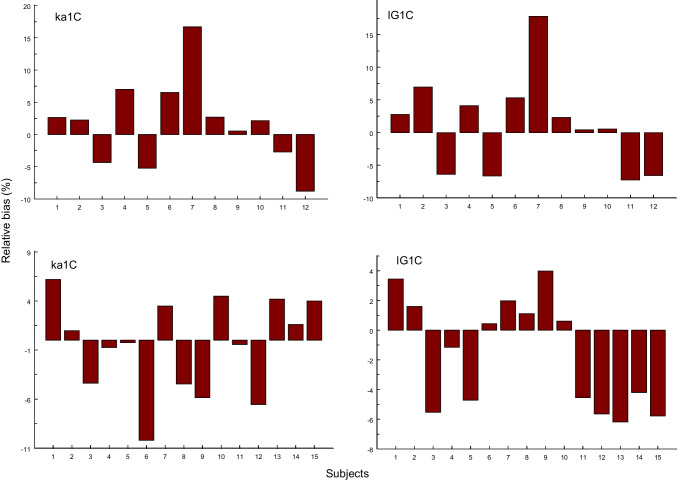
The relative biases in *AUC* estimation derived from individual fits using the ka1C model (left) and IG1C model (right) for trospium (above) and ketamine 80 mg (below).

An important and unexpected result is that the Bateman function allowed a robust estimation of the population mean value of *AUC* with biases less than 3% in 5 cases and 11% in one case (Table II). This may speak for its use as an empirical curve model in population kinetics. In contrast, the individual estimates are highly biased, but the positive and negative deviations cancel each other out (Fig. 3). However, the model fails in the estimation of *MAT*, with an underestimation between 25% and 70% in 5 cases and 6% in one case (Table II). That the replacement of first order absorption by the more realistic IG input model (IG1C) does not improve this situation, shows that the reason lies in the one-compartment approximation with its unrealistic assumption of instantaneous drug distribution throughout the body after iv injection. This suggests that the knowledge of distribution parameters together with clearance is a sine qua non for an estimation of reasonable *MAT* values. In other words, the iv data from bioavailability studies must be fitted separately in order to fix the disposition parameters when fitting the oral data. Note that the bias of the IG1C model with respect to the *AUC* population means is hardly lower than that for the Bateman function. Since the biases of *AUC* for the gamma model are in all cases higher than those of the Bateman function, it is not a useful alternative model.

Although the assessment of alternative models was based on the accuracy of parameter estimation rather than the goodness of fit, Table I shows that the true model (2IG3C) has the lowest AIC, followed by the IG1C model in 4 of the 6 cases, while the gamma model provided the worst fit.

More complex input and disposition models as the 2IG input model and the two-compartment disposition model proved not suitable for fitting of po data (without iv data); on the one hand because of overparameterization and on the other hand due to the fact that information about the distribution process gets lost (vanishing of exponential terms). Thus, if *MAT* is long compared to the mean disposition residence time (*MDRT* = *V*_*ss*_*/CL*), log-concave curves are generated (apparent one-compartment distribution) [5]. While the Batman function is an appropriate empirical model for log-concave functions, it is not useful as a mechanistic model. Note that the *MAT* values for these drugs/formulations lay between 8 h and 13 h, with *MAT*/*MDRT* ratios of about 0.6 for trospium and R-ketamine, and 1 for propiverine. Since *MAT > MDRT* does not hold in these cases, it is not surprising that individual curves could not be adequately fitted to a Bateman function (Fig. 2).

The results reveal the problems in estimating *MAT* from po data alone. Thus, as the absolute bioavailability *F* can be only estimated when iv data are available, the same holds also for *MAT*. Yet one may argue that this conclusion was based on only three pharmacokinetic studies. However, it is clear *a priori* that the oral *C*(*t*)-curve is the result of the input and disposition process: Using *C*(*t*)-data after po administration we can estimate the mean body residence time (*MBRT*) from the first moment of the curve

8 $$MBRT=\int_0^{\infty } tC(t) dt/ AUC$$

(e.g., *MBRT* = 1/*k*_*a*_ + 1/*k*_*e*_ for the ka1C model and MBRT = *a*/*b* for the gamma model), and

*MAT* is determined by.

9 $$MAT= MBRT\hbox{--} MDRT$$

Since *MDRT* can be estimated only from iv data, it is impossible to estimate *MAT* when no iv reference data are available. This problem remains also when a more complex input function improves the fit. This fact has not been recognized in some recently proposed models of drug absorption that are based on a one-compartment disposition model, e.g. [18–20]. Interestingly, the shortcomings of using the one-compartment approach to analyze drug absorption kinetics have been pointed out already 55 years ago [21]. Note also that recently the finite absorption time concept was proposed as an alternative to the one-compartment model [22].

In contrast to *MAT*, the area under the curve (*AUC*) after po administration can be estimated with any empirical model that fits the data. While the biases of *AUC* estimates (Table II and Fig. 3) are only characteristic for these drugs/formulations, analogous results may be obtained for other slowly absorbed drugs or extended release formulations. While the results obtained from such case studies cannot be simplistically generalized, at least the assumption that the Bateman function may be useful for fitting oral *C*(*t*)-data could not be disproved.

## Conclusions

When no iv data are available, the Bateman function appears to be useful for estimating the population mean value of *AUC* after po administration. The low bias in the mean values is in contrast to the highly biased individual estimates of *AUC*. The results of this study reemphasize the fact that insight into the absorption process can be only gained when also iv reference data are available. Whereas this is well accepted for bioavailability, there is less agreement regarding the estimation of parameters of absorption kinetics (e.g. *MAT*). It is clear from theory and our results that the one- compartment approximation is not useful for this purpose.

### Supplementary Information

ESM 1 (DOCX 108 kb)
