# Supplementary material for: Is the One-Compartment Model with First Order Absorption a Useful Approximation?
Source: Pharm Res. 2023 Aug 18;40(9):2147–53. doi: 10.1007/s11095-023-03582-1 (PMC10547630; doi:10.1007/s11095-023-03582-1)
Supplement: Supplementary file 1 — (DOCX 108 kb) [file 11095_2023_3582_MOESM1_ESM.docx]

**SUPPLEMENTARY MATERIAL**

Is the one-compartment model with first order absorption a useful approximation?

Michael Weiss^1^

^1^Department of Pharmacology, Martin Luther University Halle-Wittenberg, Halle,
Germany

Figure S1. Mean (± SEM) plasma concentration time curve of trospium (30 mg) immediate release tablets (n = 12) [9].

Figure S2. Mean (± SEM) plasma concentration time curve of propiverine extended release tablets, filled circles 10 mg, filled triangles 45 mg (n = 10) [10].

Figure S3. Mean (± SEM) plasma concentration time curve of R-ketamine extended release tablets, filled circles 10 mg, filled triangles 80 mg (n = 15) [11].
